# A chamber study of respiratory and autonomic responses to hydrotreated vegetable oil exhaust using ECG-derived respiration

**DOI:** 10.3389/fphys.2026.1710755

**Published:** 2026-06-03

**Authors:** Mostafa Abdollahpur, Louise Gren, Katrin Dierschke, Leo Stockfelt, Aneta Wierzbicka, Frida Sandberg

**Affiliations:** 1 Department of Biomedical Engineering, Lund University, Lund, Sweden; 2 Ergonomics and Aerosol Technology, Lund University, Lund, Sweden; 3 Occupational and Environmental Medicine, Region Skåne, Lund, Sweden; 4 Department of Occupational and Environmental Medicine, Sahlgrenska University Hospital, Gothenburg, Sweden; 5 Occupational and Environmental Medicine, School of Public Health and Community Medicine, Institute of Medicine, University of Gothenburg, Gothenburg, Sweden

**Keywords:** air pollution, cardiorespiratory coupling (CRC), ECG-derived respiration (EDR), heart rate variability (HRV), hydrotreated vegetable oil (HVO), mutual information (MUI)

## Abstract

**Background:**

Air pollution is a major public health concern, contributing to cardiovascular and respiratory diseases. One of the main pathways by which air pollution affects cardiovascular health is through dysregulation of the autonomic nervous system (ANS), which impairs cardiac control. Hydrotreated vegetable oil (HVO) is a renewable fuel with unknown toxicity in comparison to conventional diesel, and the effects of HVO exhaust on autonomic and respiratory regulation remain unclear.

**Objective:**

This study investigates the physiological responses to HVO exhaust exposure by assessing heart rate variability (HRV), ECG-derived respiration (EDR) and cardiorespiratory coupling (CRC).

**Methods:**

A controlled exposure study was conducted with 19 healthy volunteers (10 males, 9 females, aged 20–55) who underwent four different exposure scenarios in a randomized, double-blind design: filtered air, filtered air with NaCl particles, HVO exhaust with an aftertreatment system, and HVO exhaust without aftertreatment. ECG signals were continuously recorded, and HRV, EDR, and CRC features were extracted. A linear mixed model was used to assess time- and exposure-dependent changes.

**Results:**

No statistically significant differences in HRV, EDR, or CRC features were found between exposure scenarios.

**Conclusion:**

In this study, short-term exposure to HVO exhaust, corresponding to EU OELs for one workday, was not associated with statistically significant changes in the ECG-derived autonomic, respiratory, or cardiorespiratory-coupling markers assessed in healthy volunteers at the studied pollutant concentrations.

## 1 Introduction

Air pollution represents one of the most pressing global public health challenges, contributing significantly to the global burden of non-communicable diseases, particularly cardiovascular conditions (Fuller et al., 2022). Among airborne pollutants, fine particulate matter (PM_2_._5_) is a leading environmental risk factor, linked to approximately 4.2 million premature deaths globally in 2019, with 70% attributed to cardiovascular diseases (CVDs) (Miller et al., 2024). Long-term exposure to PM_2_._5_ is strongly associated with increased cardiovascular morbidity and mortality, particularly in low- and middle-income countries (Guo et al., 2023).

Short-term cardiovascular effects of air pollution are mediated through multiple overlapping mechanisms, including systemic inflammation, oxidative stress, endothelial dysfunction, and thrombogenicity (Rajagopalan et al., 2018). Among these, autonomic nervous system (ANS) imbalance has emerged as a critical pathway, often reflected by changes in heart rate variability (HRV). Exposure to airborne particles can activate pulmonary irritant receptors and sensory afferents in the airways, triggering reflexive autonomic responses that alter heart rate and vascular tone (Franklin et al., 2015). This vagally mediated pathway may lead to increased parasympathetic withdrawal, sympathetic activation, or both, contributing to acute cardiovascular instability. HRV and arterial stiffness are key markers of these autonomic changes and have been consistently associated with pollutant exposure in both ambient and controlled exposure studies (Ganji et al., 2024; Jeong et al., 2024).

Cardiorespiratory coupling (CRC), the physiological interaction between the cardiovascular and respiratory systems, is also regulated by the ANS and plays a role in maintaining cardiovascular stability and efficient gas exchange (Dick et al., 2014). Pollution-induced disturbances in CRC are hypothesized based on evidence that air pollutants, including diesel exhaust, can impair respiratory sinus arrhythmia (RSA) and baroreflex sensitivity (Taylor-Clark, 2020; Abdul-Rahman et al., 2024). Diesel exhaust exposure has been associated with autonomic imbalance, reduced HRV, and increased cardiovascular risk (Taylor-Clark, 2020; Stockfelt et al., 2022; Kuntic et al., 2023). To the best of our knowledge, CRC analysis has not previously been applied in chamber-based air pollution exposure studies. Given that CRC is considered a sensitive marker of physiological stress (Abreu et al., 2023), applying this approach to pollutant exposure may offer new insights into early autonomic dysfunction.

In recent years, hydrotreated vegetable oil (HVO), a paraffinic biofuel derived from hydrogenated vegetable oils or animal fats, has been promoted as a cleaner alternative to petroleum diesel due to its low particulate and polycyclic aromatic hydrocarbon (PAH) emissions (Zeman et al., 2019; Goberdhan, 2023; Bendtsen et al., 2020). However, the biological effects of HVO exhaust remain poorly characterized. Concerns persist regarding the formation of potentially toxic intermediates during combustion and their possible impact on respiratory and cardiovascular systems.

To address the health implications of renewable fuels such as HVO, controlled exposure studies provide an important framework for assessing short-term physiological responses under standardized conditions. Chamber studies reduce confounding by controlling environmental variability and allow for repeated measures designs that are well suited for detecting subtle physiological changes in autonomic, respiratory, or vascular function.

In 2019, we conducted a controlled chamber exposure study at Lund University to investigate the short-term health effects of HVO emissions (Gren et al., 2022). The study included four exposure scenarios; HVO exhaust with aftertreatment, HVO exhaust without aftertreatment, filtered clean air, and filtered air with dry NaCl particles. Previous analyses focused on inflammatory responses and showed that, at the studied concentrations, HVO emissions caused limited physiological changes in healthy volunteers (Krais et al., 2021; Scholten et al., 2021). Cardiovascular analyses revealed some alterations in vascular compliance but no consistent HRV changes when assessed in selected 5-minute segments (Marc-Derrien et al., 2022).

In the present work, we track cardiorespiratory dynamics by extracting ECG-derived respiration (EDR) and combining it with HRV to assess CRC analysis. This approach enables a detailed evaluation of how HVO emissions influence autonomic function and the synchronization between cardiac and respiratory rhythms. To evaluate these effects, we apply a linear mixed model (LMM) framework that accounts for both fixed effects of exposures and inter-individual variability.

## 2 Methodology

### 2.1 Data acquisition

The study involved 19 healthy volunteers (10 males and 9 females, aged 20–55 years), who were exposed to four different exposure scenarios, each lasting three hours. The study was approved by the Swedish Ethical Review Authority (registration Dnr: 2019-03320) and performed in accordance with the Declaration of Helsinki, including obtaining written informed consent from all subjects. The exposure levels were designed to be close to, but below, the current EU 8-hour occupational exposure limits (OELs) relevant for diesel exhaust, including NO, NO2, BTEX, PAHs (such as naphthalene and benzo[a]pyrene), and elemental carbon (EC). The exposures were conducted in a controlled 22 m^3^ stainless steel chamber at Lund University, Sweden, with an air exchange rate of four changes per hour.

As described earlier by (Gren et al., 2022), the study was designed to analyze the effect of the two HVO exposures (C, D) in comparison to filtered air (A). Exposure to aerosolized dry NaCl (B) was added to analyze the effects of exposure to soluble and non-toxic particles (NaCl) in comparison to filtered (A) air as well as to two HVO exposures. The vehicles used for two HVO exposures were manufactured in 2019 and complied with the current non-road engine EU emission standards. The wheel loader without an aftertreatment system allowed creating exposure to airborne particles, including EC and nitrogen oxide (NOx), whereas the vehicle with aftertreatment system provided exposure consisting mainly of NOx as particles were removed efficiently. The exposure scenarios are summarized below:

- Filtered air, PM1 ≈ 1 µg/m³ (PM1 particulate matter with diameter< 1 µm).
- Filtered air with dry NaCl, PM2.5 ≈ 120 µg/m³ (PM2.5 particulate matter with diameter< 2.5 µm).
- Emissions from a wheel loader with an aftertreatment system running on HVO (PM1 ≈ 1 µg/m³, NO = 2.0 ppm, NO2 = 0.7 ppm).
- Emissions from a wheel loader without an aftertreatment system running on HVO (PM1 ≈ 90 µg/m³, EC = 54 µg/m³, NO = 3.4 ppm, NO2 = 0.6 ppm).

A more detailed chemical characterization of the exposure conditions has been reported previously by (Gren et al., 2022). Briefly, the HVO exposure without aftertreatment contained higher particle mass and elemental carbon than the HVO exposure with aftertreatment as listed above. In the HVO exposure without aftertreatment, particle-phase PAHs were 43 ± 3 ng/m^3^ and gas-phase PAHs were 850 ± 48 ng/m^3^, while the corresponding concentrations in HVO exposure with aftertreatment were 0.3 ± 0.7 ng/m^3^ and 97 ± 11 ng/m^3^, respectively. The sum of BTEX was 7.9 ± 2.5 *µ*g/m^3^ in the HVO exposure without aftertreatment and 1.7 ± 0.3 *µ*g/m^3^ in HVO exposure with aftertreatment. In HVO exposure with aftertreatment, the concentrations of particle-phase PAHs, gas-phase PAHs, and BTEX were relatively low and close to filtered air levels, i.e., 0.2 ± 0.2 ng/m^3^, 116 ± 29 ng/m^3^, and 1.3 ± 0.1 *µ*g/m^3^, respectively. These differences in particle and gas phase characteristics are relevant when interpreting the physiological findings, as particle concentrations, elemental carbon, and organic compounds such as PAHs and BTEX may contribute differently to autonomic and respiratory responses.

The study followed a double-blind, randomized exposure design in which participants were exposed to the different scenarios on separate occasions in a random order. Exposures were conducted in the morning hours (between 9 a.m. and 12 p.m.) on different days of the week, with a minimum one-week interval between each session to avoid carryover effects. The study was carried out during the fall season to reduce the risk of allergens affecting the participants. Participants were instructed to abstain from caffeine on the morning of the sessions and from alcohol for at least 24 hours before each session. They were also asked to maintain similar routines throughout the study to ensure consistency (Gren et al., 2022).

The temperature in the chamber was 26 ± 1 °C with a relative humidity of 33 ± 4%. Participants were exposed in groups of up to four individuals, sitting quietly during the three-hour exposure. Background noise levels in the chamber were kept between 42 and 46 dB, primarily due to the ventilation system and surrounding equipment. Before the exposure sessions, participants underwent a baseline visit to become familiar with the chamber and medical procedures, reducing potential biases due to unfamiliarity. Threelead ECG was continuously recorded during the experimental protocol at a sampling frequency of 1 kHz using a custom-made medical device, CardioHolter v6.2, developed at Kaunas University of Technology, Lithuania; HRV and EDR were extracted from these continuous ECG recordings. Additionally, respiration rate was measured once for each volunteer during 15 minutes in proximity to one of the exposure scenarios using a respiratory inductance plethysmograph (Nox T3 breathing belt, Nox Medical, ResMed) and tidal volume was measured using forced oscillation technique while subjects breathed through a mouthpiece prior to each exposure, these measurements were used for dose calculations.

### 2.2 ECG signal processing

To ensure reliable respiratory signal monitoring, ECG lead II was selected for analysis due to its optimal alignment with the body’s longitudinal axis, which enhances the detection of respiratory-induced variations in thoracic impedance (Wan et al., 2024). This lead measures the electrical potential difference between the right arm and left leg electrodes, providing a clear view of the heart’s electrical activity and pronounced respiratory signal modulation. The data obtained from Lead II served as the foundation for subsequent preprocessing and feature extraction.

To reduce high-frequency noise, a fourth-order Butterworth low-pass filter with a cutoff frequency of 50 Hz was applied to the ECG signal. After that, the ECG signal was divided into consecutive five-minute segments. Following R-peak detection, a QRS template was derived for each five-minute segment by averaging aligned QRS segments within a window extended from 100 ms before to 200 ms after the R-peak, resulting in a total duration of 300 ms. To enable exclusion of ectopic beats and beat detections caused by non-physiological artifacts, QRS segments with a correlation to the QRS template below 0.9 were labeled as non-normal heartbeats.

### 2.3 Heart rate variability analysis

The RR interval series, denoted as *RR*(*i*), was constructed as shown in Equation 1:

(1) $$RR\left(i\right)=t_{E}\left(i\right)-t_{E}\left(i-1\right)$$

where *t_E_*(*i*) represents the time of the *i^th^* R-peak.

The normal-to-normal (*NN*) interval series represents the *RR* intervals between successive normal heartbeats. *RR* intervals that immediately preceded or followed non-normal beats, as well as intervals deviating more than 20% from the average of the 50 preceding *RR* intervals, were excluded to obtain the *NN* interval series.

Classical time-domain and frequency-domain HRV features were then extracted for each five minute segment. Time-domain features, namely mean *NN* interval (*NN_mean_*), standard deviation of *NN* intervals (*SDNN*), root mean square of successive differences (*RMSSD*), and standard deviation of successive differences (*SDSD*), were calculated to characterize the variability in heart rate.

To enable frequency-domain analysis, the *NN* intervals were resampled to 4 Hz using cubic spline interpolation. The resampled signal was then detrended by removing its mean using a moving average filtering approach, in which the moving average, calculated over a 100-sample window, was subtracted from the resampled signal to eliminate low-frequency variations below 0.04 Hz. The resulting signal quantifying heart rate variations is denoted $S_{HRV}\left(n\right)$. Power spectral density was then estimated from $S_{HRV}\left(n\right)$ using the fast fourier transform (FFT) to calculate the power in the low-frequency (LF, 0.04-0.15 Hz) and high-frequency (HF, 0.15-0.4 Hz) bands. Additionally, the *LF/HF* ratio was calculated as shown in Equation 2:

(2) $$LF/HF=\frac{P_{LF}}{P_{HF}}$$

where *P_LF_* and *P_HF_* represent the power in the LF and HF bands, respectively. The relative power in the LF and HF bands was also estimated compared to the total power (*LF_norm_* and *HF_norm_*).

### 2.4 ECG-derived respiration

An ECG derived respiration signal was obtained by analyzing the slope range of QRS complexes as proposed by (Kontaxis et al., 2019). This approach quantifies variations in QRS morphology, which are assumed to correspond to respiratory activity, by calculating the difference between the maximum and minimum derivative within the QRS interval (*y*(*n*)), as expressed in Equation 3:

(3) $$r\left(i\right)=\underset{n}{\max}\{y_{i}^{\prime }\left(n\right)\}-\underset{n}{\min}\{y_{i}^{\prime }\left(n\right)\},$$

where *i* represents the beat number, and $y_{i}^{\prime }\left(n\right)=y_{i}\left(n\right)-y_{i}\left(n-1\right)$.

To enhance the reliability of the EDR signal, values deviating more than 5 times the average median deviation from the median *r*(*i*) in the analyzed segment were identified as outliers and replaced through linear interpolation.

Following outlier correction, *r*(*i*) was resampled to a uniform frequency of 4 Hz using cubic spline interpolation. Subsequently, detrending was applied by removing the mean and using a moving average filter with a 100-sample window. This step eliminates low-frequency components, effectively isolating respiratory-related fluctuations in the signal. The resulting respiration signal was denoted $S_{EDR}\left(n\right)$.

The $S_{EDR}\left(n\right)$ signal was evaluated for reliability and respiratory feature extraction using frequency domain analysis. The power spectral density was computed to identify the dominant respiratory frequency, *F_R_*, defined as to the largest peak within the respiratory range (0.1-0.4 Hz). Signal quality was assessed by calculating the ratio of the largest peak in the respiratory range to the largest peak in the entire spectrum. Following (Kontaxis et al., 2019), the signal is considered reliable if this ratio exceeds 0.85. This procedure ensures that $S_{EDR}\left(n\right)$ accurately reflects respiratory activity. The magnitude of $S_{EDR}\left(n\right)$ is quantified by its root mean square value, as shown in Equation 4:

(4) $$A_{R}=\sqrt{\frac{1}{N}\sum_{n=1}^{N}{𝒮}_{EDR}{\left(n\right)}^{2}}$$

where *N* is the total number of samples in the 5-minute segment, i.e., 1200. This metric reflects respiratory chest movements and has been shown to correlate with tidal volume (Lázaro et al., 2024).

### 2.5 Cardiorespiratory coupling

Cardiorespiratory coupling refers to the interaction between cardiac and respiratory systems, offering insights into their physiological synchronization (Schulz et al., 2013; Dick et al., 2014). This study explores both linear and nonlinear coupling between $S_{HRV}$ and $S_{EDR}$. Note that $S_{HRV}$ and $S_{EDR}$ are both sampled at 4 Hz and aligned using a common time vector.

#### 2.5.1 Coherent cross-power spectral analysis

To evaluate the strength of the linear coupling between $S_{HRV}$ and $S_{EDR}$ we used the cardio pulmonary coupling indexes proposed in (Hilmisson et al., 2024). The cardiopulmonary coupling index at frequency *f* is calculated as shown in Equation 5 the product of cross-spectral power and coherence at each frequency *f*, integrating both the magnitude of common oscillations and their phase synchronization,

(5) $$CPC\left(f\right)=\left|P_{HRV,EDR}\left(f\right)\right|C_{HRV,EDR}\left(f\right)$$

where *P_HRV,EDR_*(*f*) is the cross-power spectral density between $S_{HRV}$ and $S_{EDR}$, representing the shared power at *f*. The cross-spectral coherence (*C_HRV,EDR_*(*f*)) between $S_{HRV}$ and $S_{EDR}$ is defined as shown in Equation 6.

(6) $$C_{HRV,EDR}\left(f\right)=\frac{|P_{HRV,EDR}\left(f\right){|}^{2}}{P_{HRV}\left(f\right)P_{EDR}\left(f\right)}$$

where *P_HRV_*(*f*) and *P_EDR_*(*f*) are the auto-power spectral densities of $S_{HRV}$ and $S_{EDR}$, respectively.

The *P_HRV_*(*f*), *P_EDR_*(*f*) and *P_HRV,EDR_*(*f*) are obtained using Welch’s method with 50% overlapping.

1024-sample subwindows, corresponding to 256 seconds. Each subwindow is linearly detrended and windowed using a Hamming window to minimize spectral leakage. An FFT length of 1024 was used, yielding 512 frequency points from 0 Hz to 2 Hz corresponding to a frequency resolution of approximately 0.0039 Hz. These settings were chosen based on previously published work in CPC analysis (Thomas et al., 2005). Following the calculation of *CPC*(*f*), three indexes were derived; *CPC_LF_*quantifying cardiorespiratory coupling in the low-frequency band, *CPC_HF_* quantifying cardiorespiratory coupling in the high-frequency band, and *CPC_LF/HF_* quantifying the ratio between these. *CPC_LF_*was obtained as the mean of *CPC*(*f*) values within the 0.04 ≤ *f<* 0.15* Hz* interval whereas *CPC_HF_* was obtained as the mean *CPC*(*f*) in the 0.15 ≤ *f* ≤ 0.4 Hz interval. The ratio between *CPC_HF_*and *CPC_LF_* serves as an indicator of autonomic regulation and respiratory stability; a preponderance of power in the LF band is associated with periodic breathing patterns and sympathetic activation, while a higher HF coupling is indicative of respiratory sinus arrhythmia and vagal dominance (Hilmisson et al., 2024).

#### 2.5.2 Mutual information

Mutual information (MUI) is a statistical measure derived from information theory that quantifies the shared information or dependency between two signals. It is particularly valuable for analyzing complex physiological interactions, such as those between $S_{HRV}$ and $S_{EDR}$, as it captures both linear and nonlinear dependencies (Schulz et al., 2013).

The concept of MUI is based on Shannon entropy (*H_x_*), which measures the uncertainty or information content of a time series *x*. Shannon entropy, introduced by Shannon (Shannon, 1948), is calculated as shown in Equation 7.

(7) $$H_{x}=-\sum_{i=1}^{M}p\left(x_{i}\right){\text{log}}_{2}p\left(x_{i}\right),$$

where *p*(*x_i_*) is the probability of the *i*-th symbolic state of the time series *x*, and *M* is the total number of symbolic states. Higher entropy values reflect greater uncertainty and lower predictability. Mutual information extends this concept by considering the shared entropy between two signals. It is defined as shown in Equation 8.

(8) $${\text{MUI}}_{xy}=H_{x}+H_{y}-H_{xy},$$

where *H_x_* and *H_y_* represent the individual entropies of signals *x* and *y*, respectively, and *H_xy_* represents their joint entropy. Notably, MUI is symmetric (MUI*_xy_*= MUI*_yx_*), non-negative (MUI*_xy_*≥ 0), and bounded by min (*H_x_,H_y_*) (Cover, 1999; Steuer et al., 2002).

The amplitudes of $S_{HRV}$ and $S_{EDR}$ were discretized into *M* = 3 symbolic states using quantile-based thresholds, dividing the amplitude range into three equal-probability bins. The thresholds were set to the 0th, 33rd, 66th, and 100th percentiles of $S_{HRV}\left(n\right)$ and $S_{EDR}\left(n\right)$, respectively. Each sample was then mapped to a symbolic state such that HRV*_i_*∈ {1,2,3} and EDR*_i_*∈ {1,2,3}, depending on which interval the value fell into.

The marginal probabilities *p*_HRV_(*HRV_i_*) and *p*_EDR_(*EDR_i_*), as well as the joint probability distribution *p*_HRV_,_EDR_(*HRV_i_,EDR_i_*), were estimated from the symbolized sequences using normalized histograms. The individual and joint entropies were calculated as shown in Equations 9–11:

(9) $$H_{\text{HRV}}=-\sum_{i=1}^{3}p_{\text{HRV}}\left(HRV_{i}\right)\underset{2}{\log}p_{\text{HRV}}\left(HRV_{i}\right)$$

(10) $$H_{\text{EDR}}=-\sum_{j=1}^{3}p_{\text{EDR}}\left(EDR_{i}\right)\underset{2}{\log}p_{\text{EDR}}\left(EDR_{i}\right)$$

(11) $$H_{\text{HRV},\text{EDR}}=-\sum_{i=1}^{3}\sum_{j=1}^{3}p_{\text{HRV},\text{EDR}}\left(HRV_{i},EDR_{j}\right)\underset{2}{\log}p_{\text{HRV},\text{EDR}}\left(HRV_{i},EDR_{j}\right)$$

Because both signals were discretized using quantile-based thresholds into three equally probable symbolic states, the individual entropies *H*_HRV_ and *H*_EDR_ are expected to be equal and close to the theoretical maximum of log_2_ 3 ≈ 1.585.

Finally, the MUI between the HRV and EDR signals was computed as shown in Equation 12:

(12) $$MUI_{\text{HRV},\text{EDR}}=H_{\text{HRV}}+H_{\text{EDR}}-H_{\text{HRV},\text{EDR}}$$

This method allowed for the detection of both linear and nonlinear interactions, providing a comprehensive assessment of the degree of coupling between the cardiovascular and respiratory systems. Greater MUI values indicated stronger cardiorespiratory coupling, while smaller values reflected diminished coupling or independence.

#### 2.5.3 Statistical analysis

A linear mixed model (LMM) was fitted to the HRV, EDR and cardiorespiratory coupling trends to analyze the effects of the exposures. A LMM was utilized to account for the hierarchical structure of the data and potential correlations within subjects due to repeated measurements. LMMs are particularly suitable for datasets involving repeated measures, as they incorporate both fixed effects, representing the systematic influence of predictors (e.g., exposure type and time), and random effects, accounting for individual variability among subjects and their unique time trends (Rindskopf, 2023). The LMM used in this study is given by Equation 13.

(13) $$\begin{array}{ll} Y_{ijp} & =\beta_{0}+\beta_{t}Time_{j}+\beta_{B}\times Time_{j}+\beta_{C}\times Time_{j}+\beta_{D}\times Time_{j}+b_{0ip}+b_{1ip}Time_{j}+\in_{ijp}, \end{array}$$

where *Y_ijp_* denotes the measurement of a given feature (e.g., *P*_LF_, *P*_HF_, *LF/HF* ratio) for subject *i* at time point *j* under exposure scenario *p*; *β*_0_ is the fixed intercept representing the measurement level during the control exposure scenario A; *β_t_* captures the fixed effect of time across all exposure scenarios; *β_B_*, *β_C_*, and *β_D_* represent the additional time-trend effects for exposure scenarios B, C, and D relative to scenario A (each coefficient is zero for observations not in its corresponding scenario); *b*_0_*_ip_* and *b*_1_*_ip_* are random effects accounting for subject-specific intercepts and time slopes within each exposure scenario; and *∈_ijp_* is the residual error term. The inclusion of the exposure-specific random intercepts (*b*_0_*_ip_*) and random slopes (*b*_1_*_ip_*) allows each subject to have a unique baseline and time trend for each exposure scenario.

The random effects *b*_0_*_ip_* and *b*_1_*_ip_* are assumed to follow a multivariate normal distribution:


$$\left[\begin{array}{c} b_{0ip}\\ b_{1ip} \end{array}\right]∼N\left(\left[\begin{array}{c} 0\\ 0 \end{array}\right],\left[\begin{array}{cc} \sigma_{b_{0}}^{2} & \text{Cov}\left(b_{0},b_{1}\right)\\ \text{Cov}\left(b_{0},b_{1}\right) & \sigma_{b_{1}}^{2} \end{array}\right]\right),$$

and the residual error *∈_ijp_* is assumed to be Gaussian:


$$\in_{ijp}∼N\left(0,\sigma_{\in }^{2}\right).$$

Hence, the total variance in the response variable *Y_ijp_* can be decomposed as shown in Equation 14:

(14) $$Var\left(Y_{ijp}\right)=\sigma_{b_{0}}^{2}+\sigma_{b_{1}}^{2}+\sigma_{\in }^{2},$$

where $\sigma_{b_{0}}^{2}$ represents the between-subject variability of the feature, $\sigma_{b_{1}}^{2}$ represents the between-subject variability in the rate of change of the feature over time, and $\sigma_{\in }^{2}$ captures the within-subject variability.

Model parameters were estimated using restricted maximum likelihood, which provides unbiased estimates of variance components. To assess the significance of fixed effects, Wald tests were performed (Pinheiro and Bates, 2000).

## 3 Results

### 3.1 Heart rate variability analysis

**Figure 1:**
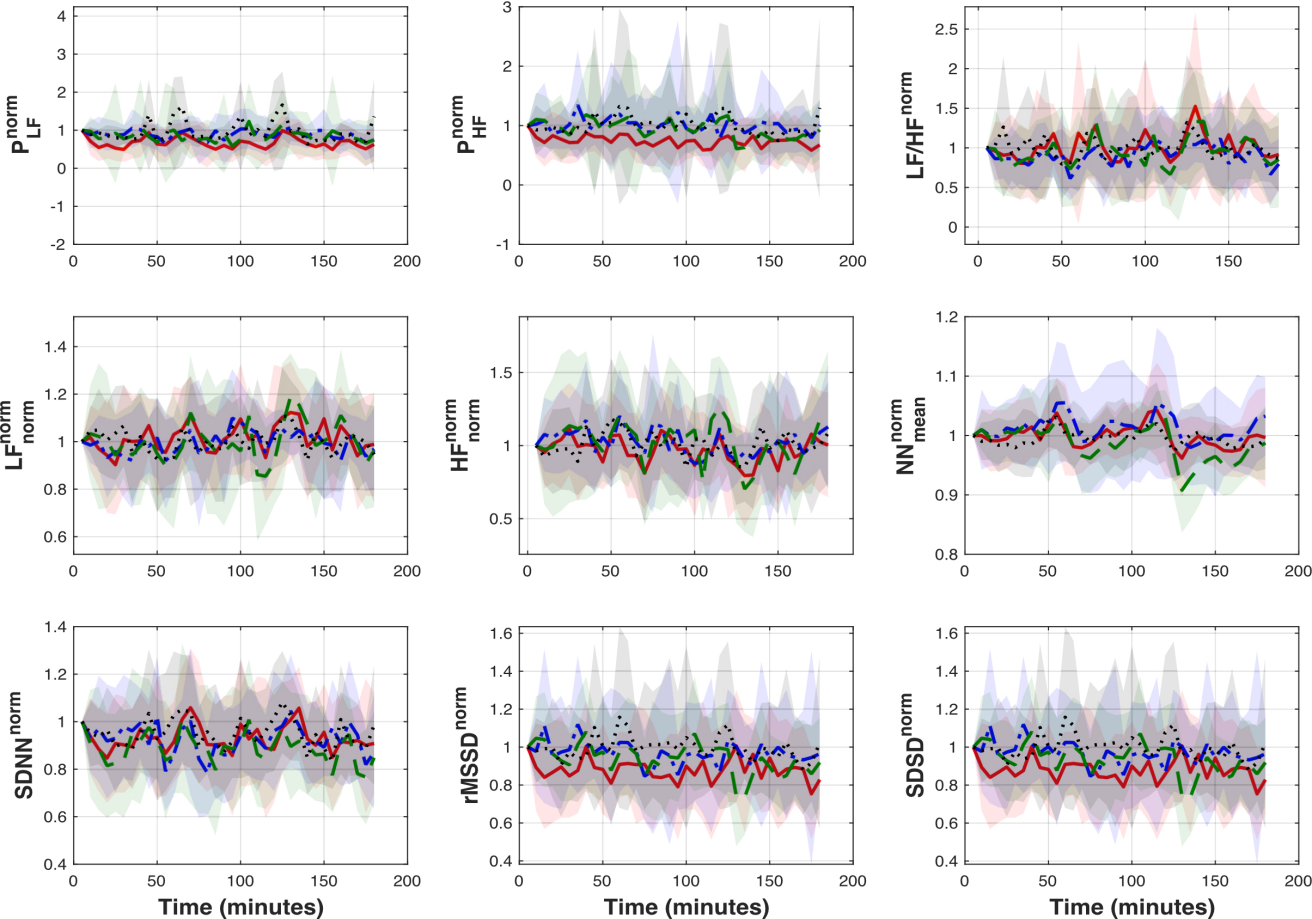
Temporal trends of normalized HRV features across all exposure scenarios. Each line represents the population mean for one exposure scenario: red (solid) for Scenario **(A)** blue (dash-dot) for Scenario **(B)** green (dashed) for Scenario **(C)** and black (dotted) for Scenario **(D)** The shaded regions indicate ±1 SD around the mean, reflecting inter-subject variability. Normalization was performed by dividing each subject’s values by their initial value (i.e., the first valid 5-minute segment) within each exposure scenario. This allows visualization of relative changes over time while accounting for individual baseline differences.

The temporal trends of the HRV features, normalized to the initial value of each exposure scenario and subject (i.e., the first valid 5-minute segment of each exposure scenario), are displayed in **Figure 1**, providing a comprehensive view of their variations over time.

To quantify these trends and assess exposure effects, the linear mixed-effect model in eq 13 were fitted separately to each HRV feature. The fixed effects analysis (**Table 1**) revealed small but significant timerelated changes (*β_t_*) for *LF*_norm_, *HF*_norm_, and *NN*_mean_. However, no consistent modulation by exposure scenario (*β_B_*, *β_C_*, *β_D_*) was observed across features.

**Table 1:** Fixed effects coefficients for HRV features.

Feature	*β*_0_	*β_t_*	*βB*	*βC*	*βD*
*PLF* (*s*2)	0.0053^∗^	−2.37 × 10^−6^	−2.67 × 10^−7^	−3.35 × 10^−6^	−1.18 × 10^−8^
*PHF* (*s*2)	0.0029^∗^	−2.80 × 10^−6^	1.50 × 10^−6^	−2.20 × 10^−6^	−5.46 × 10^−7^
*LF/HF*	2.2032^∗^	1.28 × 10^−3^	9.32 × 10^−4^	5.01 × 10^−4^	1.32 × 10^−3^
*LFnorm*	0.6103^∗^	2.66 × 10^−4^*	−2.57 × 10^−5^	−5.98 × 10^−5^	−7.87 × 10^−5^
*HFnorm*	0.3897^∗^	−2.66 × 10^−4^*	2.57 × 10^−5^	5.98 × 10^−5^	7.87 × 10^−5^
*NN_mean_*(*s*)	1.0159^∗^	−1.97 × 10^−4^*	−1.49 × 10^−5^	−1.98 × 10^−4^	2.26 × 10^−5^
*SDNN* (*s*)	0.0661^∗^	1.20 × 10^−5^	−8.44 × 10^−6^	−3.75 × 10^−5^	−4.24 × 10^−5^
*RMSSD* (*s*)	0.0611^∗^	−1.75 × 10^−5^	−1.21 × 10^−5^	−4.47 × 10^−5^	−2.08 × 10^−5^
*SDSD* (*s*)	0.0612^∗^	−1.76 × 10^−5^	−1.21 × 10^−5^	−4.47 × 10^−5^	−2.08 × 10^−5^

Significant coefficients (*p<* 0.05) are marked with an asterisk (*).

Random effects analysis confirmed marked inter-subject variability in HRV parameters (*b*_0_), as summarized in results (**Table 2**). The estimated resting heart rate, based on modeled *NN*_mean_ values (*β*_0_ + *b*_0_), ranged from approximately 51 to 70 beats per minute across subjects and exposure scenarios. Random slopes (*b*_1_) were near zero for all features, indicating stable within-subject trends over time. Residual variances ( $\sigma_{\in }^{2}$) were consistently low, further supporting model fit and reliability.

**Table 2:** Random intercepts (*b*_0_), random slopes (*b*_1_), and residual error variance $\sigma_{\in }^{2}$ for HRV features.

Feature	*b*0	*b*1	$\sigma_{\in }^{2}$
*PLF* (*s*2)	−5.00×10^−5^ ± 9.79×10^−3^	4.07×10^−8^ ± 1.09×10^−5^	3.77 × 10^−6^
*^P^HF* (*s*^2^)	−1.07×10^−5^ ± 3.71×10^−3^	1.19×10^−8^ ± 7.13×10^−6^	1.04 × 10^−6^
*LF/HF*	−2.22×10^−3^ ± 1.367	−1.05×10^−6^ ± 3.60×10^−3^	1.16 × 10^−2^
*LFnorm*	−1.39×10^−4^ ± 0.136	1.84×10^−7^ ± 3.79×10^−4^	9.97 × 10^−3^
*HFnorm*	1.39×10^−4^ ± 0.136	−1.84×10^−7^ ± 3.79×10^−4^	9.97 × 10^−3^
*NN_mean_*(*s*)	−2.53×10^−5^ ± 0.152	3.74×10^−8^ ± 3.96×10^−4^	2.31 × 10^−3^
*SDNN* (*s*)	9.33×10^−5^ ± 0.0207	−1.81×10^−7^ ± 5.90×10^−5^	1.69 × 10^−4^
*rMSSD* (*s*)	7.21×10^−5^ ± 0.0323	−1.12×10^−7^ ± 8.53×10^−5^	1.71 × 10^−4^
*SDSD* (*s*)	7.22×10^−5^ ± 0.0324	−1.12×10^−7^ ± 8.55×10^−5^	1.72 × 10^−4^

Values are presented as *mean* ± *std*.

ECG recordings were partially missing for one participant in exposure scenario A, one in exposure scenario B, three in exposure scenario C, and one in exposure scenario D. The total number of 5-minute segments per participant and exposure scenario, and the corresponding number of segments valid for HRV analysis according to the criteria outlined in Sec 2.2 and Sec 2.3 are summarized in **Table 3**; the results described above are based on these segments.

**Table 3:** Total number of 5-minute segments and the number of segments valid for HRV analysis for each participant and exposure scenario.

Subject	Exposure scenario A (Valid/Total)	Exposure scenario B (Valid/Total)	Exposure scenario C (Valid/Total)	Exposure scenario D (Valid/Total)
1	33/37	23/37	32/37	30/37
2	27/37	24/38	35/36	33/36
3	37/37	37/38	37/37	30/30
4	30/38	28/38	31/36	–
5	28/37	16/33	37/38	36/37
6	35/37	28/38	34/37	25/37
7	25/36	19/38	25/37	11/37
8	36/38	20/37	35/37	34/36
9	36/36	36/38	37/37	25/39
10	17/36	31/37	27/39	29/37
11	33/40	34/38	–	17/37
12	20/35	8/38	17/36	31/40
13	36/59	29/37	34/38	29/39
14	27/37	23/38	–	16/38
15	32/38	–	35/37	22/37
16	–	32/37	–	26/37
17	15/36	17/37	31/37	33/36
18	27/37	38/38	30/37	34/37
19	33/38	22/38	36/37	37/38

Missing recordings are shown in “–”.

### 3.2 ECG derived respiration

**Figure 2:**
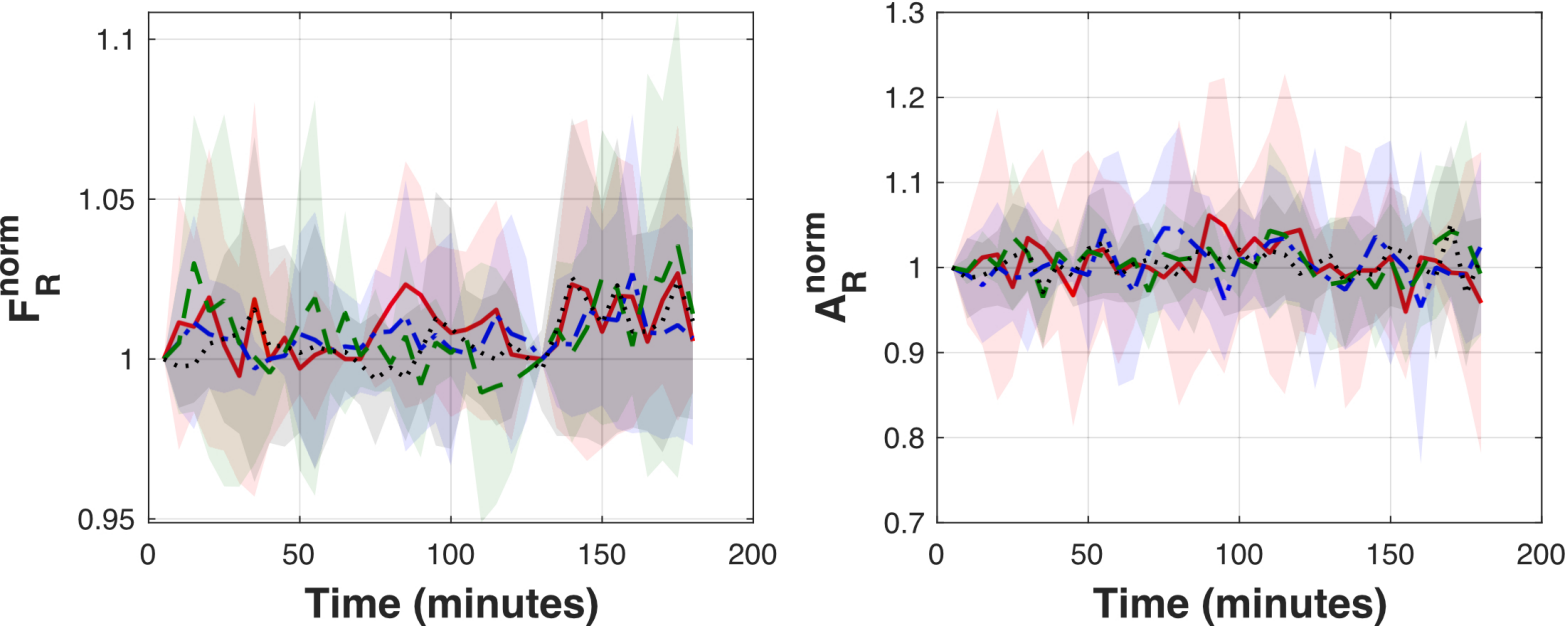
Temporal trends of normalized respiratory frequency (*F_R_*) and ECG-derived respiratory amplitude (*A_R_*) across all exposure scenarios. Each line represents the population average for one exposure scenario: red (solid) for Scenario **(A)** blue (dash-dot) for Scenario **(B)** green (dashed) for Scenario **(C)** and black (dotted) for Scenario **(D)** The shaded areas represent ±1 SD from the mean, indicating inter-subject variability. Normalization was performed by dividing each subject’s values by their initial value (i.e., the first valid 5-minute segment) within each exposure scenario. This enables comparison of relative temporal changes while accounting for individual baseline differences.

The 5-minute segments deemed valid for HRV analysis (**Table 3**) were processed for EDR extraction. The number of segments with EDR deemed reliable, as described in Sec 2.4, is reported in **Table 4**. The temporal trends of the EDR features, normalized to the initial value of each exposure scenario and subject, are displayed in **Figure 2**, providing a comprehensive view of their variations over time.

**Table 4:** Total number of 5-minute segments and number of segments valid for EDR signal extraction for each participant and exposure scenario.

Subject	Exposure scenario A (Valid)/Total	Exposure scenario B (Valid/Total)	Exposure scenario C (Valid/Total)	Exposure scenario D (Valid/Total)
1	5/37	11/37	6/37	5/37
2	7/37	15/38	22/36	22/36
3	28/37	26/38	31/37	19/30
4	20/38	14/38	19/36	–
5	18/37	8/33	22/38	13/37
6	11/37	10/38	24/37	13/37
7	10/36	9/38	12/37	7/37
8	18/38	7/37	13/37	10/36
9	25/36	29/38	19/37	9/39
10	11/36	6/37	10/39	13/37
11	23/40	15/38	–	9/37
12	11/35	4/38	7/36	11/40
13	32/59	20/37	12/38	24/39
14	21/37	20/38	–	12/38
15	27/38	–	11/37	9/37
16	–	25/37	–	18/37
17	11/36	10/37	4/37	21/36
18	13/37	20/38	16/37	12/37
19	14/38	9/38	4/37	18/38

Missing recordings are shown in “–”.

The fixed effects coefficients of the fitted linear mixed-effects model for *F_R_*and *A_R_*are summarized in **Table 5**. The time effects (*β_t_*) and their exposure scenario-specific interactions (*β_B_*, *β_C_*, *β_D_*) were small and not statistically significant, indicating that respiratory frequency and amplitude remained stable over time and across exposure scenarios.

**Table 5:** Fixed effects coefficients for EDR features.

Feature	*β*_0_	*β_t_*	*βB*	*βC*	*βD*
*F_R_*(*Hz*)	0.2592^∗^	2.61 × 10^−5^	−2.15 × 10^−6^	−1.06 × 10^−5^	2.63 × 10^−5^
*A_R_*(*a.u.*)	0.2885^∗^	−2.53 × 10^−5^	7.82 × 10^−5^	3.17 × 10^−5^	2.63 × 10^−5^

Significant coefficients (*p<* 0.05) are marked with an asterisk (*).

Random effects analysis (**Table 6**) showed random slopes (*b*_1_) were near zero, indicating stable withinsubject respiratory trends over time. Residual error variances ( $\sigma_{\in }^{2}$) were low, reflecting a good model fit with minimal unexplained variability.

**Table 6:** Random intercepts (*b*_0_), random slopes (*b*_1_), and residual error variance ( $\sigma_{\in }^{2}$) for EDR features.

Feature	*b*0	*b*1	$\sigma_{\in }^{2}$
*F_R_*(*Hz*)	−1.18×10^−4^ ± 4.17×10^−2^	5.68×10^−7^ ± 5.55×10^−5^	7.99 × 10^−4^
*A_R_*(*a.u.*)	6.93×10^−5^ ± 3.19×10^−2^	9.59×10^−7^ ± 4.29×10^−5^	1.43 × 10^−3^

Values are presented as *mean* ± *std*.

**Figure 3:**
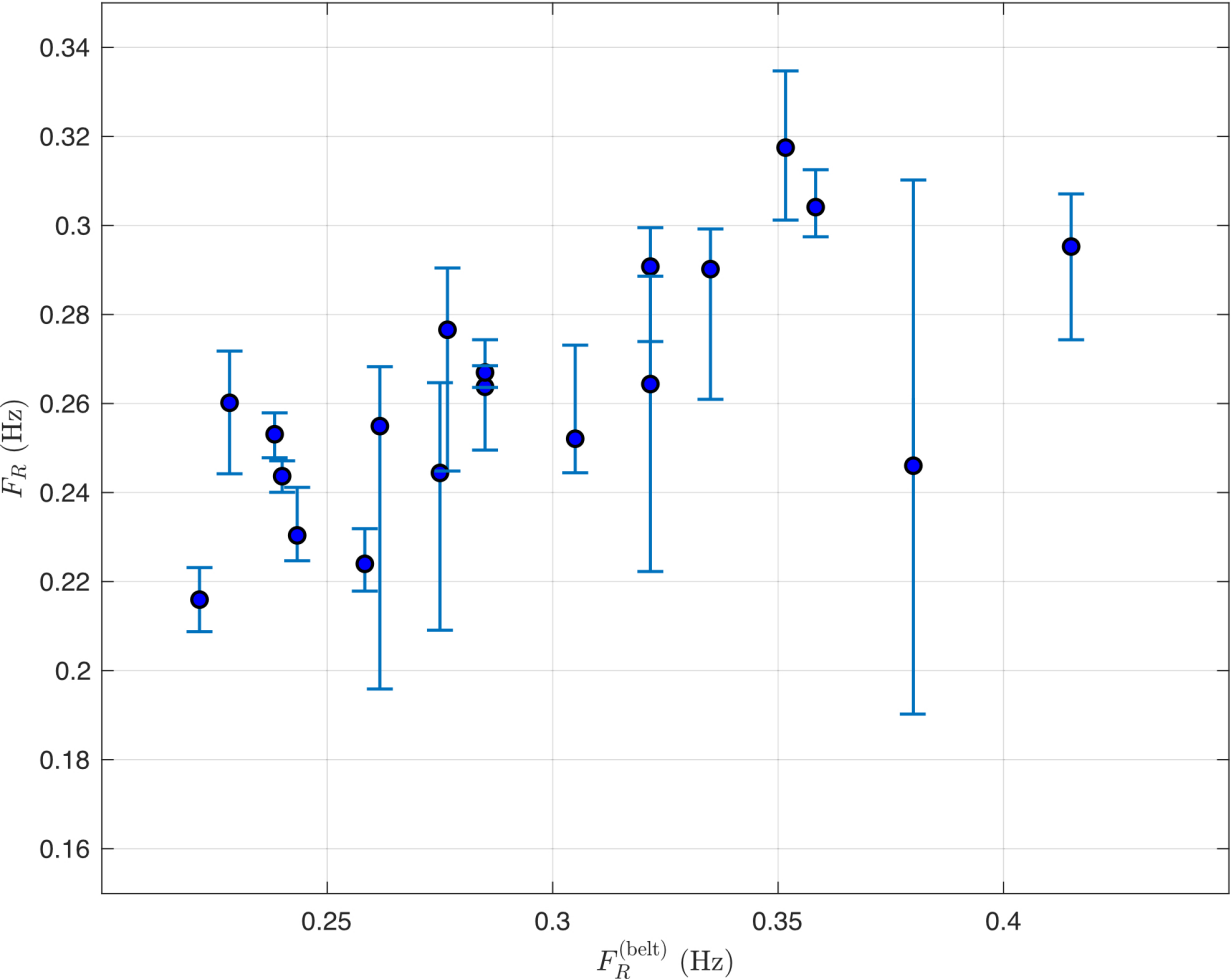
Scatter plot showing the (dot) median and (bars) 25th and 75th percentile of *F_R_*versus the corresponding belt-derived respiration rate ( $F_{R}^{\left(\text{belt}\right)}$) for each subject.

The estimated resting respiratory rate (i.e., *β*_0_ + *b*_0_) ranged approximately from 0.18 to 0.34 Hz, corresponding to 11 to 21 breaths per minute. To evaluate the accuracy of EDR, the median and interquartile range of *F_R_*over all valid segments for each subject was compared with reference measurements obtained using a respiratory inductance plethysmograph, $\;F_{R}^{\left(\text{belt}\right)}$, recorded once for each volunteer during a 15-min period in proximity to one of their exposure sessions (**Figure 3**). A moderate-to-strong positive correlation was found between median *F_R_* and belt-derived respiration rate, with a Pearson correlation coefficient of *r* = 0.69 (*p* = 0.0011), indicating that the EDR method reliably captured individual differences in breathing rate. Most subjects showed good agreement, with small individual errors. The mean squared error (MSE) ranged from 1.0 × 10^−7^ to 0.0105 Hz^2^ across subjects.

**Figure 4:**
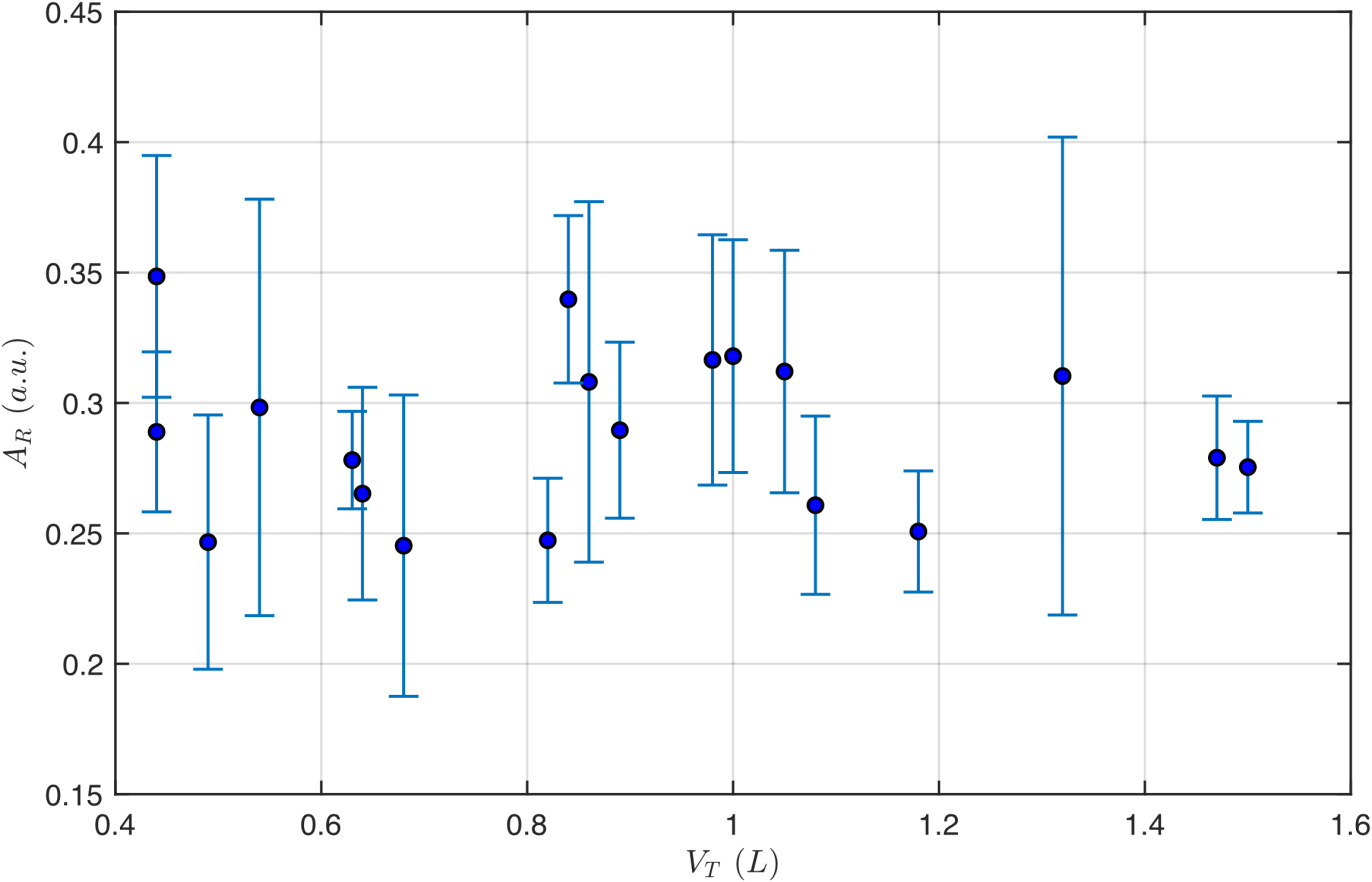
Scatter plot showing the (dot) median and (bars) 25th and 75th percentile of *A_R_*versus the corresponding tidal volume *V_T_*for each subject.

Further, we compared *A_R_*and the average of the tidal volumes measured using forced oscillation technique prior to each exposure (*V_T_*; **Figure 4**). No correlation was found between *A_R_*and *V_T_*(Pearson’s *r* = −0.040, *p* = 0.871), indicating that *A_R_*cannot be used as a surrogate for tidal volume.

### 3.3 Cardiorespiratory coupling

**Figure 5:**
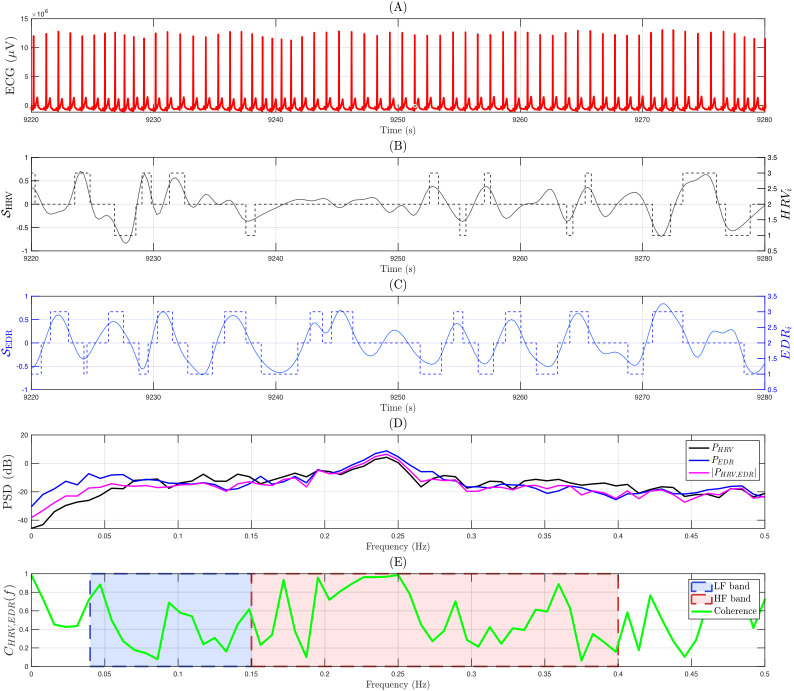
Visualization of cardiac and respiratory signals extracted from a 5-minute ECG segment from Subject 3, exposure scenario A. **(A)** preprocessed ECG signal over a representative 60-second window from this segment. **(B, C)** normalized HRV and EDR signals (S*_HRV_*and S*_EDR_*), respectively, overlaid with their corresponding symbolic sequences (*HRV_i_*and *EDR_i_*). **(D)** power spectral densities of HRV and EDR (*P_HRV_*(*f*) and *P_EDR_*(*f*)), and their cross-power spectral density (|*P_HRV,EDR_*(*f*)|). **(E)** coherence spectrum *C_HRV,EDR_*(*f*) between HRV and EDR, with shaded regions indicating the low-frequency (LF: 0.04–0.15 Hz) and high-frequency (HF: 0.15–0.4 Hz) bands.

**Figure 5** presents representative data from a five minute segment of one participant (Subject 3, exposure scenario A) to illustrate the processing steps involved in CRC analysis. **Figure 5A** displays the preprocessed ECG signal over a 60-second window. In **Figures 5B, C**, $S_{HRV}$ and $S_{EDR}$ are shown alongside their corresponding symbolic sequences *HRV_i_*and *EDR_i_*. **Figure 5D** shows the power spectral densities *P_HRV_*(*f*) and *P_EDR_*(*f*), as well as the magnitude of their cross-power spectral density |*P_HRV,EDR_*(*f*)|. **Figure 5E** shows the coherence spectrum *C_HRV,EDR_*(*f*), with shaded regions marking the low-frequency and high-frequency bands used to derive *CPC_LF_*, *CPC_HF_*, and *CPC_LF/HF_*.

**Figure 6:**
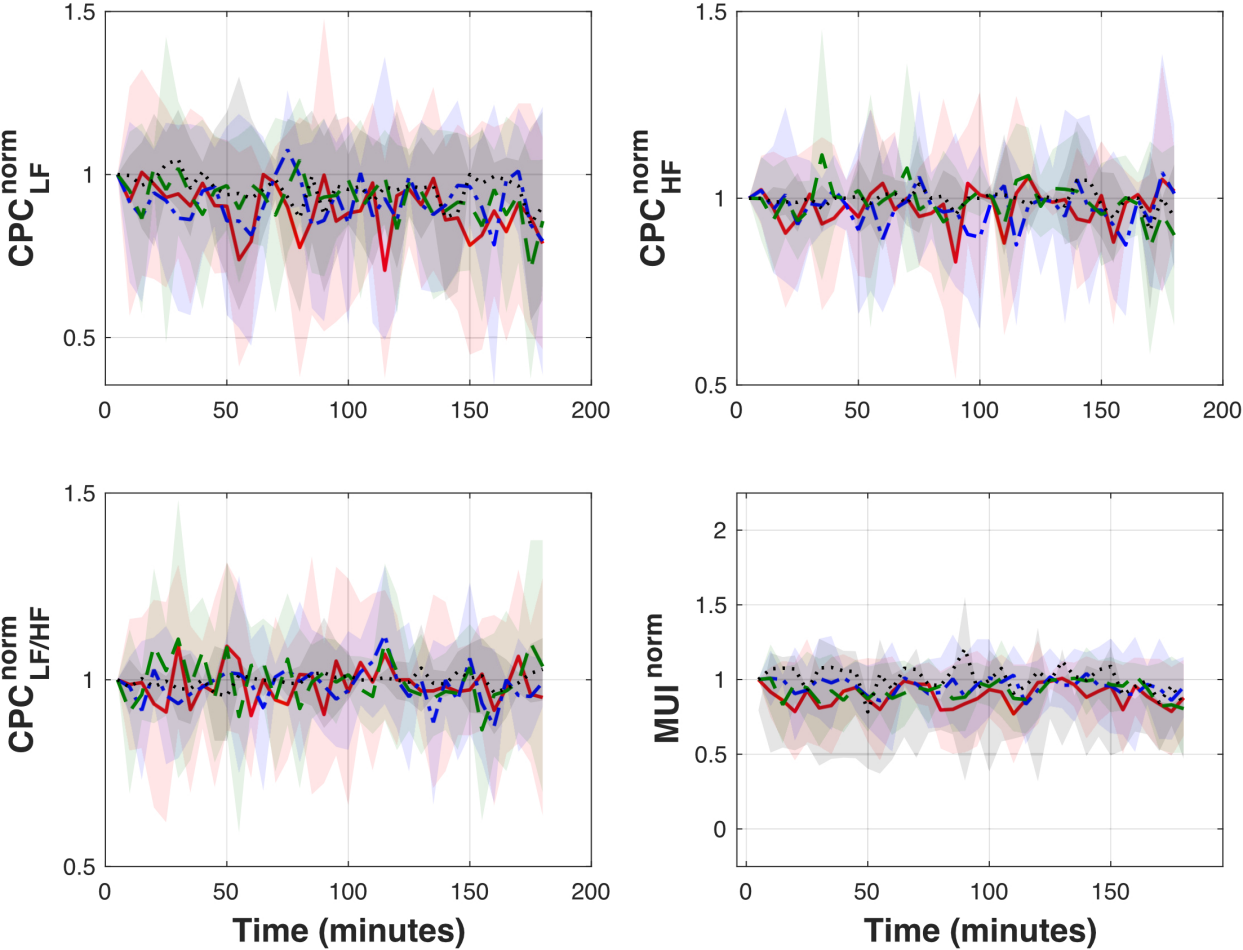
Temporal trends of normalized *CPC_LF_*, *CPC_HF_*, *CPC_LF/HF_*, and *MUI* across all exposure scenarios over time. Each line represents the population mean for one exposure scenario: red (solid) for Scenario **(A)** blue (dash-dot) for Scenario **(B)** green (dashed) for Scenario **(C)** and black (dotted) for Scenario **(D)** The shaded regions indicate ±1 SD around the mean, reflecting inter-subject variability. Normalization was performed by dividing each subject’s values by their initial value (i.e., the first valid 5-minute segment) within each exposure scenario. This approach allows for comparison of relative temporal changes across individuals while accounting for baseline variability.

The temporal trends of *CPC_LF_*, *CPC_HF_*, *CPC_LF/HF_*, and *MUI* normalized to the initial value for each exposure scenario and subject are displayed in **Figure 6**, providing a comprehensive view of their variations over time.

Linear mixed-effects models were fitted to each CRC feature to evaluate time and exposure effects (**Table 7**). None of the time-related coefficients (*β_t_*) or exposure interaction terms (*β_B_*, *β_C_*, *β_D_*) reached statistical significance, suggesting that CRC features remained largely stable and were not systematically influenced by exposure scenario or time.

**Table 7:** Fixed effects coefficients for CPC features.

Feature	*β*_0_	*β_t_*	*βB*	*βC*	*βD*
*MUI* (*n.u.*)	0.1012^∗^	−3.11×10^−5^	4.70×10^−5^	−3.92×10^−5^	−1.80×10^−5^
*CPC_LF_*(*n.u.*)	0.1615^∗^	1.12×10^−5^	−8.38×10^−6^	−3.57×10^−5^	−1.52×10^−5^
*CPC_HF_*(*n.u.*)	0.1222^∗^	7.34×10^−6^	−3.54×10^−6^	−2.78×10^−5^	1.06×10^−5^
*CPC_LF/HF_*(*n.u.*)	1.9399^∗^	−1.94×10^−4^	−2.48×10^−4^	9.44×10^−5^	4.02×10^−4^

Significant coefficients (*p<* 0.05) are marked with an asterisk (*).

Random effects analysis (**Table 8**) revealed generally low inter-subject variability in baseline levels (*b*_0_) for most features, except *CPC_LF/HF_* with standard deviations of 1.33. The larger variability in *CPC_LF/HF_*is expected given its nature as a ratio metric and its sensitivity to fluctuations in autonomic balance. Random slopes (*b*_1_) were consistently near zero, indicating stable within-subject trajectories over time. The largest residual variance was again observed in $CPC_{LF/HF}\left(\sigma_{\in }^{2}=0.14\right)$, reflecting greater within-subject variability compared to the other CRC features.

**Table 8:** Summary of random intercepts (*b*_0_), random slopes (*b*_1_), and residual error variance ( $\sigma_{\in }^{2}$) for CRC features.

Feature	*b*0	*b*1	$\sigma_{\in }^{2}$
*MUI* (*n.u.*)	−2.88×10^−4^ ± 4.96×10^−2^	9.47×10^−8^ ± 8.40×10^−5^	4.91×10^−4^
*CPC_LF_*(*n.u.*)	4.06×10^−4^ ± 4.48×10^−2^	2.08×10^−7^ ± 3.67×10^−5^	3.75×10^−4^
*CPC_HF_*(*n.u.*)	2.58×10^−4^ ± 4.27×10^−2^	1.14×10^−7^ ± 1.88×10^−5^	3.58×10^−4^
*CPC_LF/HF_*(*n.u.*)	8.64×10^−3^ ± 1.329	−2.23×10^−6^ ± 4.82×10^−4^	1.38×10^−1^

Values are presented as *mean* ± *std*.

## 4 Discussion

This study evaluated the short-term physiological effects of exposure to HVO exhaust on autonomic and respiratory regulation in healthy adults, using heart rate variability, ECG-derived respiration, and cardiorespiratory coupling measures. Across all exposure scenarios, including HVO with and without aftertreatment, no statistically significant differences were observed in the HRV, EDR, or CRC outcomes, indicating stability in autonomic nervous system regulation in response to these short-term exposures.

The HRV results showed minimal temporal variation across exposure scenarios. A small but statistically significant increase in *LF_norm_*and a corresponding decrease in *HF_norm_*was observed over time, as reflected in the *β_t_*coefficients (**Table 1**). However, these shifts were consistent across all exposure conditions as indicated by the non-significant *β_B_*, *β_C_*and *β_D_*(**Table 1**). This suggests that the variations in *HF_norm_*and *LF_norm_*are likely attributable to normal intra-day variation or circadian influences, rather than exposure-induced changes. These findings emphasize the importance of accounting for time-of-day effects in short-term exposure studies, as failing to do so could result in spurious positive or negative effects. The absence of shifts in HRV between exposure scenarios supports the conclusion that short-term HVO exposure, at the tested concentrations, did not produce measurable perturbations in autonomic function. This is also consistent with the results from our previous analysis of the present study data based on selected 5-minute ECG segments (Marc-Derrien et al., 2022).

This lack of exposure-specific autonomic effects contrasts with findings from previous chamber studies using similar protocols but different particle types. For example (Stockfelt et al., 2022), reported a significant reduction in *P_HF_*, suggesting an increase in parasympathetic activity, during exposure to diesel exhaust particles at substantially higher concentrations (300 µg/m³), both alone and in combination with road traffic noise. In contrast, noise alone had no effect on HRV. Similarly (Hagerman et al., 2014), examined HRV responses to nano-sized indoor particles and found divergent autonomic effects depending on the particle source: *P_HF_*increased during candle smoke exposure, but decreased during exposure to terpene and ozone reaction products. These results demonstrate that HRV responses can vary considerably with particle composition and toxicity, even under highly standardized conditions. Compared to these studies, the lack of exposure dependent changes observed in the present study suggest that the autonomic effects of HVO emissions may be weaker, or that the specific particle composition elicits a less detectable response in healthy young adults.

The absence of detectable changes in HRV, EDR, and CRC in the present study may therefore reflect the specific particle and gas phase properties of the HVO exposures used here, including the relatively low particle and gas phase concentrations in exposure to HVO with aftertreatment. However, the present study was not designed to isolate the physiological effects of individual chemical constituents, and therefore no firm conclusion can be drawn regarding which components of HVO exhaust are most relevant for autonomic or respiratory regulation.

Another possible explanation for the absence of significant autonomic responses in the current study is the relatively young and healthy study population. As argued in (Stockfelt et al., 2022), individuals with resilient cardiovascular and autonomic systems may be less sensitive to short-term pollutant exposures, particularly when tested in resting, controlled conditions. While direct evidence is limited, some support for this hypothesis can be found in previous chamber studies reporting more pronounced effects in older participants or those with pre-existing vulnerabilities (Bräuner et al., 2007; Forchhammer et al., 2012). The high degree of physiological compensation in healthy adults may therefore mask subtle autonomic shifts that would be more detectable in older or more susceptible populations. Future studies could explore whether age-related changes in autonomic regulation modify sensitivity to exhaust-related exposures, particularly under real-world stress or activity conditions.

The ECG derived respiratory frequency (*F_R_*) and amplitude (*A_R_*), remained stable throughout the exposure sessions as indicated by the non-significant *β_t_*, *β_B_*, *β_C_*, and *β_D_*in (**Table 5**), suggesting that the respiratory rhythm was not substantially modulated during any exposure scenario. A moderate-tostrong correlation between *F_R_*and reference respiratory belt measurements was observed across subjects (**Figure 3**), indicating that EDR captured meaningful individual variation in breathing rate. It should be noted that the respiratory belt measurement was not obtained during the exposures, and therefore direct validation was not feasible. The respiratory amplitude, *A_R_* is a subject-specific relative measure of chest movements, that has been shown to correlate to changes in tidal volume (Lázaro et al., 2024). It is important to emphasize that *A_R_*is not a validated proxy for tidal volume as also indicated by our results (**Figure 4**). Although it reflects respiration-related variations in ECG morphology, the relationship to absolute ventilation volume is indirect and influenced by individual physiology, electrode placement, and signal quality. This distinction is particularly relevant for exposure studies that estimate deposited particle dose using models such as MPPD (multiple-path particle dosimetry model) (Asgharian et al., 2001), where both respiratory rate and tidal volume are required inputs to simulate regional deposition in the lungs. While *F_R_*derived from EDR appears to be a reliable surrogate, *A_R_* cannot substitute for direct tidal volume measurements. In the context of the current HVO exposure study, estimates of deposited dose based on MPPD modeling were performed using belt-derived tidal volume and respiratory frequency values acquired once for each subject, as reported by Gren et al (Gren et al., 2022). The limited time-variation in the EDR derived respiratory parameters in the present study suggest that this approach is robust.

Cardiorespiratory coupling metrics, including both linear (*CPC_LF_*, *CPC_HF_*, *CPC_LF/HF_*) and nonlinear (MUI) indices, also exhibited no significant temporal or exposure-related changes, as indicated by *β_t_*, *β_B_*, *β_C_*, and *β_D_* in the linear mixed-effects models (**Table 7**). Cardiorespiratory coupling has not previously been assessed in chamber-based exposure studies; therefore, direct comparison with earlier work is not possible. The CPC metrics applied in this study were selected for their sensitivity to transient cardiorespiratory coupling at resting heart rates and their established role in sleep research ( (Thomas et al., 2005; Al Ashry et al., 2021)). We complemented this with a normalized, time-averaged mutual information (MUI) metric, which offers a robust scalar index of both linear and nonlinear dependencies, is invariant to monotonic transformations, and is less affected by interindividual differences in ECG morphology or respiration amplitude. Unlike time-lagged or sliding-window approaches used in earlier studies ((Pompe et al., 1998; Hoyer et al., 2002)), our implementation estimated MUI over fixed 5-minute segments, prioritizing stable group-level comparisons over temporal detail.

A variety of other linear and nonlinear CRC measures have been proposed (Schulz et al., 2013). Coherence and transfer functions capture frequency-specific synchronization but are restricted to linear effects; Granger causality extends this by inferring directionality but relies on strong stationarity assumptions. Informationtheoretic measures such as MUI can detect both linear and nonlinear dependencies, though at the cost of greater computational demands. In this broader context, CPC emphasizes frequency-domain linear coupling, whereas MUI provides a complementary time-domain perspective by quantifying overall shared information. Each method has distinct strengths and limitations; future work may benefit from integrating multiple indices (e.g., time-resolved MI or symbolic transfer entropy) to provide a more comprehensive characterization of cardiorespiratory coupling under pollutant exposure.

While no acute physiological effects in HRV, EDR, or CRC metrics were detected across exposure scenarios, these results establish reference values for physiological stability during short-term HVO exposure under controlled conditions in healthy adults. However, the small sample size and limited exposure duration constrain the ability to detect subtle effects or to generalize findings to broader populations. These findings should not be interpreted as evidence of safety under real-world conditions, where exposure levels, durations, and individual susceptibilities may vary considerably. Instead, they highlight the utility of this methodological framework for future studies aiming to detect both immediate and delayed responses to inhaled pollutants.

A further limitation of the present study is that HRV, EDR, and CRC are indirect markers of autonomic and respiratory regulation. Although these ECG-derived measures provide continuous, non-invasive information during the exposure sessions, they do not replace direct physiological measurements of respiratory mechanics, ventilation, blood pressure, or autonomic reflex function. Continuous direct measurements of these outcomes were not available during the exposure scenarios. Therefore, the present findings should be interpreted as indicating no detectable exposure-related changes in the ECG-derived autonomic, respiratory, and cardiorespiratory-coupling markers assessed here, rather than as definitive evidence of absence of physiological effects. Future controlled exposure studies could be strengthened by simultaneous continuous physiological measurements.

The relatively small sample size is an important limitation of the present study as it reduces the ability to detect subtle physiological effects or to generalize the findings to broader populations. However, the randomized, double-blind, crossover design increased statistical efficiency by allowing each participant to serve as his or her own control, thereby reducing between-subject confounding. In addition, the use of repeated five-minute ECG segments across each three-hour exposure session provided multiple observations per participant and exposure condition. The linear mixed-effects model was selected to account for this repeated-measures structure by including both fixed effects of time and exposure-specific time trends, as well as subject- and exposure-specific random intercepts and slopes. This approach accounts for individual baseline differences and subject-specific temporal trajectories, reducing the influence of random variation in a small cohort. Nevertheless, the study may have been underpowered to detect small or subclinical effects, and the findings should therefore be interpreted as indicating no detectable changes in the assessed ECG-derived markers under the studied exposure conditions, rather than definitive evidence of absence of physiological effects.

The lower valid/total ratios observed for some participants and exposure scenarios are another limitation of the present study. These lower ratios were mainly due to conservative ECG signal-quality criteria applied before HRV analysis. Five-minute segments were excluded when ECG noise, motion-related artifacts, unreliable R-peak detection, ectopic or non-normal beats, or abnormal RR intervals prevented reliable feature extraction. In particular, QRS complexes with a correlation below 0.9 with the segment-specific QRS template were labeled as non-normal, and RR intervals adjacent to non-normal beats or deviating more than 20% from the average of the preceding 50 RR intervals were excluded. Thus, lower valid/total ratios reflect strict quality control rather than systematic exclusion related to a specific exposure scenario. While this conservative approach increased the reliability of the extracted HRV and EDR features, it reduced the number of valid segments for some participants and may have reduced statistical power and temporal coverage.

Moreover, it remains possible that acute physiological responses occurred at subclinical levels undetectable through HRV or CRC metrics alone. Complementary analyses from the same chamber exposure study by Krais et al (Krais et al., 2021). assessed inflammatory and oxidative stress biomarkers. While no exposure-related changes were found in inflammatory protein expression or urinary 8-oxodG levels, a significant increase in urinary 4-HNE-MA, an oxidative stress marker, was observed following exposure to HVO exhaust without aftertreatment. Notably, this effect was measured three hours postexposure, highlighting the importance of capturing both concurrent and delayed responses when assessing pollutant-induced health effects. These findings suggest that different physiological and molecular endpoints may reflect distinct underlying mechanisms with varying time courses, such as rapid autonomic modulation versus slower biochemical stress responses. Integrating continuous functional measures with post-exposure biomarker analyses may therefore be necessary to fully characterize the acute and subacute health effects of air pollution.

In addition to the outcome-specific findings, this study demonstrates the feasibility of continuously extracting respiratory patterns, and cardiorespiratory coupling metrics from ECG recordings using a non-invasive and unobtrusive setup. This supports the use of EDR and CRC as practical tools for realtime physiological monitoring in environmental exposure studies. Continuous ECG-based assessment enables more temporally resolved analysis of pollutant effects, potentially revealing subtle or transient physiological changes that may be missed by conventional biomarker snapshots. Such methods hold promise for broader applications in environmental health research, including occupational exposure surveillance and individualized pollutant risk assessment, where early detection and personalized monitoring of physiological responses to pollutant exposure could offer substantial benefits.

## 5 Conclusion

This randomized crossover chamber exposure study investigated the short-term effects of HVO exhaust on ECG-derived markers of autonomic and respiratory regulation in healthy adults. Across all exposure scenarios, no statistically significant differences were detected in HRV, ECG-derived respiration patterns, or cardiorespiratory coupling metrics. These findings suggest that under these exposure concentrations, short-term HVO exhaust exposure was not associated with detectable alterations in the ECG-derived autonomic, respiratory-frequency, or cardiorespiratory-coupling markers assessed. However, because these markers are indirect and the study included a relatively small group of healthy adults, the results do not exclude subtle, delayed, or subclinical physiological effects, particularly in susceptible populations or under different exposure conditions.

## Data Availability

The raw data supporting the conclusions of this article will be made available by the authors, without undue reservation.
